# Spectral Region Optimization and Machine Learning-Based Nonlinear Spectral Analysis for Raman Detection of Cardiac Fibrosis Following Myocardial Infarction

**DOI:** 10.3390/ijms26157240

**Published:** 2025-07-26

**Authors:** Arno Krause, Marco Andreana, Richard D. Walton, James Marchant, Nestor Pallares-Lupon, Kanchan Kulkarni, Wolfgang Drexler, Angelika Unterhuber

**Affiliations:** 1 Center for Medical Physics and Biomedical Engineering, Medical University of Vienna, Waehringer Guertel 18-20, 1090 Vienna, Austria; 2 IHU Liryc, University of Bordeaux, INSERM U 1045, CRCTB, F-33000 Bordeaux, France

**Keywords:** Raman spectroscopy, myocardial infarction, cardiac fibrosis, principal component analysis, support vector machine, collagen, tissue classification, nonlinear regression, spectral band selection

## Abstract

Cardiac fibrosis following myocardial infarction plays a critical role in the formation of scar tissue and contributes to ventricular arrhythmias, including ventricular tachycardia and sudden cardiac death. Current clinical diagnostics use electrical and structural markers, but lack precision due to low spatial resolution and absence of molecular information. In this paper, we employed line scan Raman microspectroscopy to classify sheep myocardial tissue into muscle, necrotic, granulated, and fibrotic tissue types, using collagen as a molecular biomarker. Three spectral regions were evaluated: region A (600–2960 cm^−1^), region B (600–1399 cm^−1^ and 1751–2960 cm^−1^), and region C (1400–1750 cm^−1^), which includes the prominent collagen-associated peaks at 1448 cm^−1^ and 1652 cm^−1^. Linear and nonlinear principal component analysis (PCA) and support vector machines (SVMs) were applied for dimensionality reduction and classification, with nonlinear models specifically addressing the nonlinearity of collagen formation during fibrogenesis. Histological validation was performed using Masson’s trichrome staining. Raman bands associated with collagen in region C consistently outperformed regions A and B, achieving the highest explained variance and best class separation in both binary and multiclass PCA models for both linear and nonlinear approaches. The ratio of collagen-related peaks enabled stage-dependent tissue characterization, confirming the nonlinear nature of fibrotic remodeling. Our findings highlight the diagnostic potential of collagen-associated Raman bands for characterizing myocardial fibrosis. The proposed PCA-SVM framework demonstrates robust performance even with limited sample size and has the potential to lay the foundation for real-time intraoperative diagnostics.

## 1. Introduction

Sudden cardiac death (SCD) remains a major cause of mortality worldwide, with myocardial infarction (MI) being a principal underlying condition [1]. MI initiates a complex sequence of tissue remodeling events involving ischemic injury, inflammatory response, fibroblast proliferation, and extracellular matrix (ECM) reorganization [2]. Healthy myocardium consists of homogeneous bundles of cardiomyocytes embedded in an ECM—a complex network of proteins primarily composed of collagen. Following MI, a cascade of structural and molecular remodeling initiates collagenous scar formation, replacing dead cardiomyocytes. Within the first week, necrosis takes place, where inflammatory cells invade the region of the MI for the phagocytosis of necrotic myocardial cells [3]. In the subsequent granulation phase, fibroblasts deposit loosely arranged collagen fibers in sprouted capillaries. Granulation progressively transforms into dense hypocellular compact fibrotic scar tissue with dilated thin-walled vessels over weeks to a couple of months. Over time, these processes result in structurally and molecularly heterogeneous myocardial regions with impaired conduction and altered excitability [4]. These changes foster the development of arrhythmogenic substrates capable of sustaining life-threatening arrhythmias, including ventricular tachycardia (VT) and ventricular fibrillation (VF) [5,6]. Despite decades of research, many individuals who succumb to SCD had no prior clinical indicators of high arrhythmic risk, underscoring the limitations of current risk stratification strategies [7]. Existing clinical approaches for evaluating arrhythmogenic risk predominantly rely on electrical and structural markers derived from surface or intracardiac electrograms, echocardiography, and magnetic resonance imaging [8,9]. However, these tools remain constrained by limited spatial resolution and do not provide direct insight into the underlying molecular composition of myocardial tissue [10,11]. As such, key determinants of arrhythmic vulnerability—such as local heterogeneity in fibrotic content, collagen organization, or metabolic alterations—are often undetected [12]. The ability to resolve and characterize these tissue-level features at high resolution and with molecular specificity could improve our understanding of post-infarct remodeling and provide a more robust basis for identifying high-risk substrates [13]. Consequently, there is growing interest in developing complementary diagnostic strategies that integrate molecular and tissue compositional measurements with conventional electrophysiological techniques. These multimodal approaches may ultimately support the development of refined diagnostic frameworks for arrhythmia risk stratification and advance personalized clinical decision-making in post-MI care.

In this context, label-free and non-invasive optical imaging techniques operating with high spatial resolution such as Raman spectroscopy (RS) have shown considerable promise in biomedical diagnostics extending far beyond simple characterization [14,15]. Spontaneous RS, with its capability to detect metabolic and molecular changes, has already proven to be useful in cardiovascular research by its sensitivity to subtle changes in cardiac tissue and can be applied in both microscopic and endoscopic settings [16]. RS enables differentiation between cardiomyocytes and cardiac fibroblasts, offering insights into the stages of scar formation [17,18]. Collagen, as a central biomarker for fibrosis, can be identified via RS, allowing the characterization of ischemic tissue and tracking of fibrillogenesis [19,20,21]. While spectral differences in the Raman signal are most prominent during early remodeling stages, the evolving collagen composition can still provide valuable molecular information to distinguish and classify into non-fibrotic, intermediate, or fully fibrotic myocardium.

The interpretation of RS data often still lacks standardization and requires sophisticated analysis. Principal component analysis (PCA) is a widely used dimensionality reduction method as it is a powerful tool in biomedical applications to reduce spectral dimensionality and identify relevant features [22]. Tissue examination can be simplified by reducing the analysis to the important principal components (PCs). However, standard PCA assumes linear relations, which may not adequately model nonlinear biological processes such as fibrotic scarring [23]. For example, the mRNA levels of collagen types I and III increase significantly during the early post-infarction phase and subsequentially stabilize, whereas collagen accumulation follows a nonlinear trajectory [24,25]. In such cases, nonlinear PCA methods, including kernel PCA, may better capture the variance in spectral data [26,27,28]. In contrast to linear PCA, nonlinear PCA uses curved PCs to better reflect complex structures. Recent developments also integrate neural network-based approaches, such as autoencoders, as core means of nonlinear PCA, with similar advantages and superiority compared to linear PCA [29]. In combination with PCA, RS has also been used to generate features for machine learning (ML) classifiers such as support vector machines (SVMs), enabling tissue classification with high accuracy [30]. SVMs offer the flexibility to conduct training with linear and nonlinear discriminants depending on the linear or nonlinear nature of the observations [31].

In this paper, we explore the potential of line scan Raman microspectroscopy (LSRM) as a label-free non-invasive technique with high spatial resolution, which might support clinical diagnostics of arrhythmogenic substrates by identifying distinct myocardial tissue types. We perform tissue classification in both binary (muscle vs. fibrotic) and multiclass (including necrotic and granulated tissue) settings to reflect different stages of fibrillogenesis based on molecular tissue information. We further evaluate the performance of linear and nonlinear PCAs for dimensionality reduction and compare linear versus nonlinear SVM models for classification. In this context, we investigate the effect of nonlinear biological effects for dimensionality reduction and ML. Classification outcomes are validated with Masson’s trichrome staining (TS), and the potential of nonlinear spectral analysis in capturing the complexity of nonlinear biological processes such as in fibrotic remodeling is evaluated.

## 2. Results

### 2.1. LSRM for Fibrotic Tissue Examination

**Figure 1:**
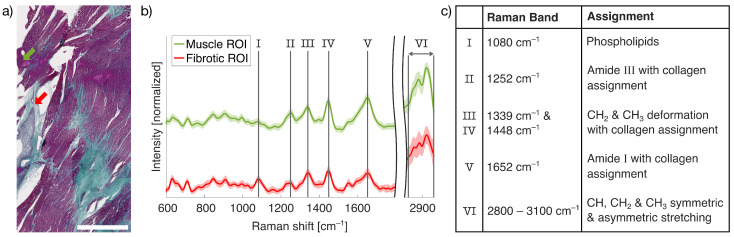
(**a**) Representative Masson’s TS section of cardiac tissue. Nuclei are stained black, normal myocardial tissue appears red, and collagen-rich fibrotic areas are blue. The purple-stained regions represent intermediate stages of fibrosis. Green and red arrows indicate the ROIs corresponding to muscle and fibrotic tissue. The white scale bar represents 1mm. (**b**) Mean Raman spectra (solid lines) and standard deviation (shaded areas) derived from the ROIs shown in (**a**). (**c**) The table summarizes the prominent Raman bands along with their corresponding biochemical assignments.

Based on histopathological evaluation, three myocardial tissue samples were classified as muscle tissue, two as necrotic, two as granulated, and two as fibrotic tissue. In general, the final stage of myocardial remodeling is characterized by dense collagen deposition forming mature scar tissue. Although the fibrotic samples showed strong collagen staining (blue) in Masson’s TS, indicative of scar formation, the presence of residual inflammatory cells as hallmarks of the preceding granulation phase prompted their pathological classification as fibrotic rather than fully scar tissue [3]. Figure 1a shows Masson’s TS, with collagen-rich fibrotic areas visualized in blue, whereas healthy muscle fibers appear in red. Necrotic tissue was identified by subtle shifts toward a more purple color, accompanied by dense accumulations of black stained inflammatory cells [32].

Upon identifying regions of interest (ROIs) within the tissue sections, LSRM was performed to capture the complete molecular fingerprint of the sample. As illustrated in Figure 1b, our LSRM system enabled the acquisition and discrimination of distinct Raman spectra from spatially adjacent muscle and fibrotic tissue. The position of the Raman acquisitions are indicated in Figure 1a by green and red arrows, representing the muscle and fibrotic ROIs, respectively. The corresponding mean spectra presented in Figure 1b reveal prominent differences between the two tissue types, with the distinguishing peaks summarized in the accompanying table in Figure 1c. The median Raman spectra of unfiltered measurements show clear differences in spectral contrast between the muscle and fibrotic samples. For better clarity, only the fingerprint region (600–1800 cm^−1^) and the CH-stretching region (2800–2960 cm^−1^) are displayed as the spectral range between 1800 cm^−1^ and 2800 cm^−1^, as silent regions lack relevant molecular tissue information. In the fingerprint region, collagen-associated bands can be clearly identified in fibrotic tissue (see table in Figure 1c). These peaks include the amide III band at 1252 cm^−1^ and CH_2_ and CH_3_ deformation modes at 1339 cm^−1^ and 1448 cm^−1^, respectively [33]. The amide I band at 1652 cm^−1^, also assigned to collagen, further highlights the key role of collagen in fibrotic cardiac remodeling. Additionally, the peak at 1080 cm^−1^, associated with phospholipids, shows the contrast between muscle and fibrotic regions [34]. Phospholipids are of particular interest in the context of MI, as they contribute significantly to the post-infarction healing process and are considered valuable markers of fibrosis [35]. In the CH-stretching region (2800–2960 cm^−1^), spectral differences are observed in the symmetric and asymmetric CH, CH_2_, and CH_3_ bands. These bands reflect lipid and protein content differences and are likely influenced by the presence of both myelinated and unmyelinated nerve fiber bundles in the tissue [36].

### 2.2. Raman Band Selection for Improved PCA

**Figure 2:**
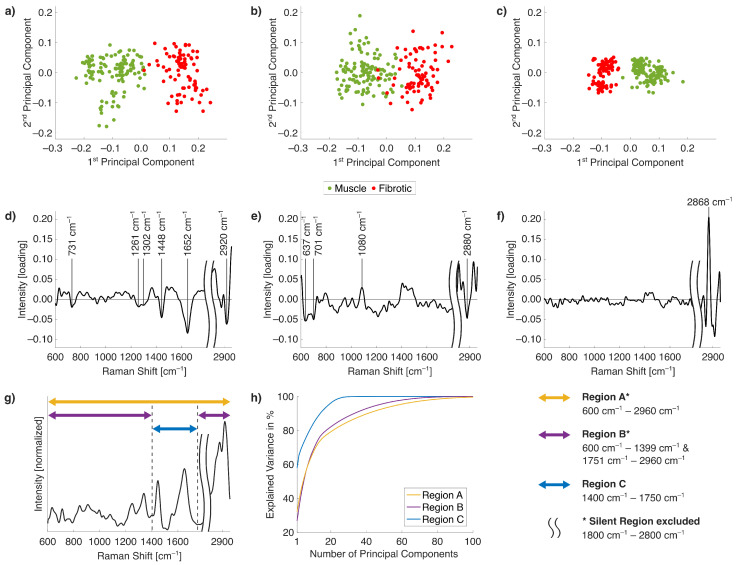
(**a**–**c**) Score plots from linear PCA applied to (**a**) spectral region A, (**b**) spectral region B, and (**c**) spectral region C, which include the prominent collagen-associated bands at $1448\;{\mathrm{cm}}^{-1}$ and $1652\;{\mathrm{cm}}^{-1}$. (**d**–**f**) Loading plots from linear PCA of spectral region A. Key Raman shifts are highlighted in first loading (**d**), second loading (**e**), and third loading (**f**). (**g**) Mean Raman spectrum of all Raman spectra indicating the spectral coverage of regions A–C. (**h**) Accumulated explained variances. Plots in (**g**,**h**) are color-coded according to the spectral region definitions used in PCA.

To assess the influence of collagen-associated spectral features for tissue differentiation, three Raman spectral regions were defined: spectral region A, spectral region B, and spectral region C. These different regions are illustrated in the mean spectrum of all data shown in Figure 2g. Spectral region C spans from $1400\;{\mathrm{cm}}^{-1}$ to $1750\;{\mathrm{cm}}^{-1}$ and includes the two prominent collagen-associated peaks at $1448\;{\mathrm{cm}}^{-1}$ and $1652\;{\mathrm{cm}}^{-1}$. Spectral region B comprises the ranges 600–1399 cm^−1^ and 1751–2960 cm^−1^, thus excluding the silent region. Spectral region A covers the broad range of Raman bands from $600\;{\mathrm{cm}}^{-1}$ to $2960\;{\mathrm{cm}}^{-1}$, also omitting the silent region (1800–2800 cm^−1^), which does not contain significant molecular information. This region definition is consistently applied in the following analysis.

PCA was applied individually to each spectral region, and scatter plots of the score values from the first two PCs are shown in Figure 2a–c. In all three cases, the first PC was primarily responsible for class separation. Among the evaluated regions, the score plot of spectral region C yielded the clearest separation between muscle and fibrotic tissue, with the highest cluster density (Figure 2c). In contrast, PCA applied to spectral region B did not show such clear discrimination and resulted in nearly overlapping clusters of muscle and fibrotic tissue, indicating limited discriminatory power, as shown in Figure 2b. Spectral region A (Figure 2a) showed moderate separation, though sub-clustering was observed for both classes, likely reflecting the inter-sample variability rather than class differences. Class separation was comparable but slightly better than spectral region B. The plots of the first, second, and third loadings from the PCA of spectral region A are shown in Figure 2d–f. The first loading confirms that the dominant features contributing to class differentiation are the collagen-related peaks at $1448\;{\mathrm{cm}}^{-1}$ and $1652\;{\mathrm{cm}}^{-1}$. These findings support the use of region C for a focused analysis of fibrotic transformation. The peaks from the C-C stretching of proline at $731\;{\mathrm{cm}}^{-1}$ and collagen-associated amide III at $1261\;{\mathrm{cm}}^{-1}$ are less pronounced [33]. The neighboring $1302\;{\mathrm{cm}}^{-1}$ peak corresponds to twisting, wagging, and bending modes of CH_2_ associated with lipids and proteins. The most prominent features of the second loading are the peaks at $637\;{\mathrm{cm}}^{-1}$ and $701\;{\mathrm{cm}}^{-1}$ assigned to amino acid methionine. Phospholipids are indicated by the vibrational band at $1084\;{\mathrm{cm}}^{-1}$ [34]. Lipid and protein contents are visible at asymmetric CH_2_ stretching vibrations at $2880\;{\mathrm{cm}}^{-1}$, and CH_3_ stretching vibrations at $2920\;{\mathrm{cm}}^{-1}$, caused by different Raman intensities of myelinated and unmyelinated nerve fibers [36]. In the third loading, distinct contributions are located in the C-H stretching region, especially by the peak at $2868\;{\mathrm{cm}}^{-1}$, which is assigned to CH_2_ symmetric stretching of lipids. The corresponding mean Raman spectrum (Figure 2g) visualizes the Raman bands for each spectral region, while the accumulated explained variance is plotted in Figure 2h, using consistent color coding (A in yellow, B in purple, and C in blue). The curve for spectral region C exhibits the steepest rise, indicating that fewer PCs are required to explain a significant portion of the variance compared to regions A and B.

Quantitative values of explained variance for the first three PCs across all spectral regions, as well as, the cumulative variance provided by the first three PCs are summarized in Table 1. For spectral region C, the first PC alone explains 57.8% of the total variance, with the first three PCs cumulatively accounting for 68.5%. In comparison, regions A and B account for only 43.9% and 40.7%, respectively. While spectral region C results in variance explanations of the first three PCs corresponding to two-thirds the total variance, the sum variance explanations of the first three PCs for spectral region A and spectral region B are far below fifty percent of the total variance. To achieve 80% of total variance explained as a commonly accepted benchmark for PCA model adequacy, spectral region C required only eight PCs, whereas spectral region A and spectral region B required 22PCs and 18PCs, respectively. These results highlight the superior information density of spectral region C, particularly due to the presence and only focus of the strong collagen-related Raman bands at $1448\;{\mathrm{cm}}^{-1}$ and $1652\;{\mathrm{cm}}^{-1}$.

### 2.3. Nonlinear Dimensionality Reduction and ML with Collagen-Based Raman Band Selection

**Figure 3:**
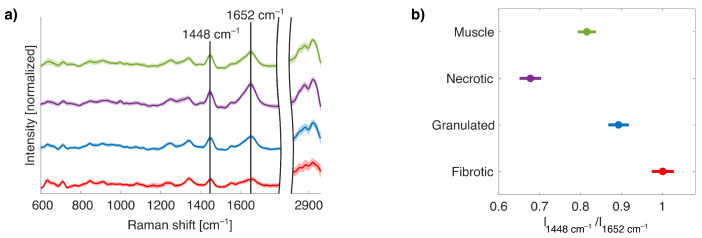
(**a**) Mean spectra of the four tissue classes: muscle, necrotic, granulated, and fibrotic tissue. Shaded areas indicate the standard deviation across all spectra within each class. (**b**) Intensity ratios of the Raman bands at $1448\;{\mathrm{cm}}^{-1}$ and $1652\;{\mathrm{cm}}^{-1}$, calculated for each individual spectrum within the respective classes. The color scheme in (**a**) corresponds to that in (**b**).

Building on the findings from the previous section, PCA based on spectral region C demonstrated better performance compared to spectral region A or B in distinguishing muscle and fibrotic tissue, primarily due to higher explained variances. However, since fibrotic remodeling is a nonlinear biological process, binary classification is insufficient to fully capture transitions over time. To address this, a four-class model was introduced, comprising muscle, necrotic, granulated, and fibrotic tissue classes, enabling a more detailed investigation of collagen-related nonlinear spectral changes over the entire wavenumber range during fibrotic scarring. As shown in Figure 3a, the mean Raman spectra of all samples reveal stage-dependent molecular changes. The necrotic class exhibited an increased collagen-associated amide I peak at $1652\;{\mathrm{cm}}^{-1}$ relative to the collagen-associated peak at $1448\;{\mathrm{cm}}^{-1}$, suggesting early collagen involvement. In contrast, granulated tissue displayed reduced peak intensities, especially at the $1652\;{\mathrm{cm}}^{-1}$ peak, resembling muscle tissue more than necrotic tissue. The trend continued for fibrotic tissue, where both peaks showed diminished contrast. To support these observations, a peak intensity ratio analysis was performed using the $1448\;{\mathrm{cm}}^{-1}/1652\;{\mathrm{cm}}^{-1}$ ratio for all spectra, followed by a one-way analysis of variance (ANOVA). The results (Figure 3b) showed statistically significant differences (p<0.05) across all class comparisons. Interestingly, the necrotic class had the lowest mean ratio (0.68), indicating a dominance of the $1652\;{\mathrm{cm}}^{-1}$ vibrational mode. Granulated and fibrotic tissues exhibited higher mean ratios, with values of 0.89 and 1.00, while muscle tissue showed a slightly reduced mean value of 0.82. Ratios are not coupled to the stage of fibrogenesis. These findings emphasize the nonlinear nature of collagen-related Raman spectral changes during scar maturation.

**Figure 4:**
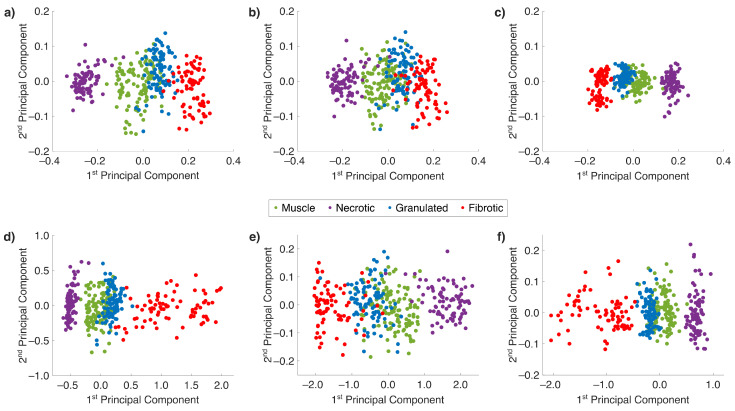
PC score plots derived from linear and nonlinear PCAs applied to different Raman spectral regions. (**a**–**c**) Linear PCA models applied to (**a**) spectral region A, (**b**) spectral region B, and (**c**) spectral region C. (**d**–**f**) Nonlinear PCA models trained with two PCs applied to (**d**) spectral region A, (**e**) spectral region B, and (**f**) spectral region C.

Given the nonlinear progression of fibrotic remodeling, standard PCA, which assumes linear relationships inside the data set, may not be sufficient. To address this potential limitation, advanced nonlinear PCA based on an autoencoder architecture was applied, as described in the *Materials and Methods* section. Both linear and nonlinear PCAs were computed using two PCs to facilitate direct comparison in a two-dimensional score plot. Figure 4a–c represent scatter plots from linear PCA models for spectral regions A, B, and C, respectively, while Figure 4d–f show corresponding plots from nonlinear PCA. In both approaches, the first PC aligns with class separation and the second PC shows roughly orthogonal behavior. Among all models, spectral region C (see Figure 4c,f) again produces the most compact and distinguishable clusters, with moderate class overlap in both linear and nonlinear cases. In contrast, spectral regions A (see Figure 4a,d) and B (see Figure 4b,e) yielded less distinct separation, especially among muscle, necrotic, and granulated tissue. Both linear and nonlinear PCAs resulted in class-dependent spreading within the groups, most notably in the fibrotic samples. In spectral region B, both linear and nonlinear PCAs presented in Figure 4b and Figure 4e, respectively, showed substantial overlap between tissue classes, and even an inverted class trend was observed between the linear and nonlinear score plots, which may be attributed to the inherent signal ambiguity in linear PCA and neural-network-based principles curves in nonlinear PCA [37,38]. However, flipping the PC sign does not change the intrinsic information.

Explained variances for the first two PCs of the PCA models in Figure 4 are summarized in Table 2. Across all spectral regions, nonlinear PCA slightly outperformed linear PCA, achieving higher variance with two PCs. Again, spectral region C achieved the highest total explained variance (78.7% using nonlinear PCA vs. 77.3% using linear PCA), reinforcing the superior discriminative power of the collagen-associated peaks at $1448\;{\mathrm{cm}}^{-1}$ and $1652\;{\mathrm{cm}}^{-1}$. The significant impact of the collagen-associated peaks is confirmed by the results in spectral region A, which accounts for a greater explained variance than spectral region B.

To optimize PCA-based classification, permutation analysis was used to determine the maximum number of significant PCs per spectral region. Significance was accepted at p<0.05, resulting in 3 PCs for spectral region C, 15PCs for region A, and 16PCs for region B. Based on these findings, both linear and quadratic SVM models were trained using three PCs for spectral region C and fifteen PCs for regions A and B, accordingly. Since PCA essentially aligns the PCs to the maximum variance in the data set, outliers may cause misleading explanations of the variance [39,40]. Therefore, the root mean square error (RMSE) was calculated for each spectral region as mentioned in the Supplemental Materials Section 1, to obtain an absolute measure about the model performance [41]. The RMSE represents the difference between original spectral data and reconstructed spectral data based on the PCA model. The reconstruction was performed using the same number of PCs as those employed during SVM training accounting for approximately 80% of the explained variance. The classification performance was evaluated across all four classes as presented in Table 3 in addition to the number of used PCs and the corresponding explained variance. Higher polynomial order or Gaussian kernels were not included to lower the risk of overfitting for the given moderate data amount. A balanced training set was provided. In all cases, linear SVM outperformed its quadratic counterpart, achieving better specificity and sensitivity values. The smallest performance difference between linear and quadratic SVM was noted for spectral region C, highlighting the robustness of the collagen-dominant features. Notably, the highest total sensitivity (90.9%) and total specificity (97.0%) were achieved using linear SVM with spectral region A. However, the best-performing quadratic SVM model was trained on spectral region C, achieving 89.1% sensitivity and 96.4% specificity. Across all models, specificity consistently exceeded sensitivity, independent of the spectral region. While nonlinear kernels improve performance, their sensitivity to data overlapping may cause adverse effects [42].

**Figure 5:**
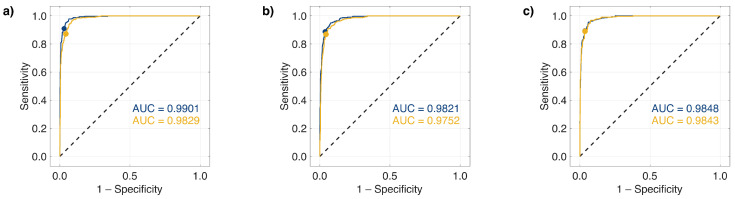
ROC curves for SVM models trained using different Raman spectral regions: (**a**) spectral region A, (**b**) spectral region B, and (**c**) spectral region C. Linear and quadratic SVM models are indicated in blue and yellow, respectively.

The receiver operating characteristic (ROC) curves in Figure 5 further illustrate classifier performance. Linear SVM models consistently produced higher area under the ROC curve (AUC) values compared to their quadratic counterparts for each spectral region. Again, spectral region C yielded the most balanced and accurate model, with minimal discrepancy between the linear and nonlinear classifiers.

For a more comprehensive analysis of spectral region optimization, two additional regions were defined: D (600–1750 cm^−1^) and E (1750–2960 cm^−1^), where region D covers the fingerprint region including the collagen-associated peaks at $1448\;{\mathrm{cm}}^{-1}$ and $1652\;{\mathrm{cm}}^{-1}$ similar to region C, and region E covers the C-H stretching region starting from $1750\;{\mathrm{cm}}^{-1}$ and excluding the silent region similar to regions A and B. The corresponding analysis is provided in the Supplemental Materials. Linear PCA results for the binary dataset, along with relevant explanations, are presented in Figure S2 and Table S1. Both linear and nonlinear PCA analyses of the quadruple dataset, with the corresponding variance explanations, are shown in Figure S3 and Table S2. To support the RMSE calculations reported in Table S3, the mean spectra with standard deviations are illustrated in Figure S4.

## 3. Discussion

Cardiac fibrosis plays a central role in structural and electrophysiological remodeling of the heart, with collagen being a key contributor to the reorganization of the ECM following injury or disease, regardless of the underlying cause [43]. In this work, we focused on the analysis of the two dominant collagen-associated Raman bands at $1448\;{\mathrm{cm}}^{-1}$ and $1652\;{\mathrm{cm}}^{-1}$ to investigate the progression of cardiac fibrotic scarring. As illustrated in Figure 1, these peaks offer insight into the molecular changes that accompany fibrogenesis. Previous work by Pioppi et al. [44] and de Campos Vidal et al. [45] have demonstrated that variations in the structure and orientation of collagen fiber bundles can be observed through shifts and intensity modulations in the amide I (1625–1700 cm^−1^) and amide III (1200–1300 cm^−1^) bands. In healthy myocardium, the ECM consists predominantly of highly organized fibrillar collagen to provide mechanical integrity of the myocardium [2]. Following MI, fibrillar collagen in necrotic myocardial tissue undergoes degradation and fragmentation, primarily driven by matrix metalloproteinases [46]. This collagen breakdown is accompanied by fibroblast activation, initiating the synthesis of loosely organized collagen during the granulation phase, which progressively develops into the deposition of dense, disorganized collagen bundles characteristic of fibrotic remodeling [3,43]. In our analysis, changes in the ratio between the amide I band at $1652\;{\mathrm{cm}}^{-1}$ and CH_2_/CH_3_ deformation band ($1448\;{\mathrm{cm}}^{-1}$) enabled the classification of fibrotic remodeling stages such as muscle, necrotic, granulated, and fibrotic tissue shown in Figure 3. The initial decrease and subsequent increase in collagen-related ratios represent a non-trivial pattern caused by the complex interplay between collagen breakdown, remodeling, and synthesis, each of which contributes to the distinct Raman signatures. We did not observe any effect of water on our intensity ratios, as the water band at $1640\;{\mathrm{cm}}^{-1}$ does not show a detectable Raman signal across all samples. Furthermore, the collagen-associated peaks at $1652\;{\mathrm{cm}}^{-1}$ and $1448\;{\mathrm{cm}}^{-1}$ do not overlap with the water band at $1640\;{\mathrm{cm}}^{-1}$.

However, spectral deconvolution has shown the potential to differentiate between collagen type I and collagen type III, where the presence of a spectral sidepeak at $1608\;{\mathrm{cm}}^{-1}$ within the amide I region is representative of structural changes in type I collagen in fibrotic tissues [47]. While collagen type I is predominantly upregulated in MI, collagen type III is more associated and increased in ischemic cardiomyopathy [48]. Despite the overlap in Raman bands between collagen type I and III, which complicates spectral separation and increases the effort for spectral deconvolution [49,50], our study focused on the total collagen content as a universal biomarker for the identification of pathological changes to detect cardiopathology at any state in fibrotic scarring relevant for an improved outcome of evaluating arrhythmogenic risks. Determining the precise cause of fibrosis may be the target of further studies by analyzing collagen type I- and collagen type III-specific contributions.

Our dimensionality reduction by PCA applied to the different spectral regions reaffirmed the significance of the collagen-associated peaks at $1448\;{\mathrm{cm}}^{-1}$ and $1652\;{\mathrm{cm}}^{-1}$. Specifically, spectral region C (1400–1750 cm^−1^) consistently yielded the highest variance explanation and had the most compact cluster separation for the first three PCs in both binary and multiclass settings (Figure 2; Table 1) with the lowest number of accumulated PCs to explain 80% of the total variance. The first loading from spectral region A confirmed the contribution of the peaks at $1448\;{\mathrm{cm}}^{-1}$ and $1652\;{\mathrm{cm}}^{-1}$ to class discrimination. While additional collagen-related bands outside of region C do exist, they exhibit lower contrast and contribute less to variance and clustering, but still enable differentiation into muscle and fibrotic tissue, as demonstrated by the PCA explanations and clustering in the score plots shown in Figure 2 [49]. However, spectral regions A and B show less clustering, which might limit the identification of fibrotic tissue. For instance, in the context of ablation therapy, accurate classification of fibrotic tissue is essential for achieving continuous ablation lesions and preventing inhomogeneous scarring [51]. Indeed, high-resolution binary classification assisting conventional electrophysiological approaches would allow for detection and terminating viable myocytes. However, the detection of pathological tissue such as necrotic or granulated tissue, which are often found in the border zone, is also of clinical interest [12]. Residual viable myocytes persist within necrotic tissue and must be targeted to avoid electrical conduction gaps. Treating necrotic zones may accelerate the transition to dense collagenous scarring, which typically takes several weeks [52].

Our results demonstrated that RS is capable of multiclass tissue discrimination, including muscle, necrotic, granulated, and fibrotic classes. These distinctions were clearly reflected in the nonlinear progression of fibrotic scarring using intensity ratios of the collagen peaks at $1448\;{\mathrm{cm}}^{-1}$ and $1652\;{\mathrm{cm}}^{-1}$, as shown in Figure 3. Interestingly, nonlinear PCA offered only marginal improvements over linear PCA, with variance explanations increased by just 1.4% to 2.8%, depending on the spectral region (Table 2). Among the evaluated spectral regions, spectral region C consistently showed the highest variance explanations and best class separation with a moderate overlap between muscle and granulated tissue, which may be explained by comparable spectral shapes and the composition of muscle and granulated tissue, as shown in Figure 3, regardless of the PCA variant used. Previous research efforts also showed a nonlinear shaping especially for the transition from granulated to fibrotic tissue [20]. However, nonlinear PCA did not outperform linear models significantly in our dataset, even though nonlinear PCA was expected to better capture fibrotic progression. While larger sample sizes generally enhance model performance, the dataset used here was considered adequate for both linear and nonlinear PCAs, since the number of spectra exceeded the number of PCs, ensuring a reliable dimensionality reduction [29,53]. However, SVM classification further confirmed the potential to focus on spectral regions that include collagen bands and confirm collagen as an important biomarker. Prior to training, permutation analysis identified the number of significant PCs for each spectral region, 3 for region C, 15 for region A, and 16 for region B. For spectral region C, the maximum number of significant PCs was used for SVM training. In contrast, the number of significant PCs for spectral region A and B was reduced to 15 to ensure a cumulative explained variance, which is comparable to the values of spectral region C. This restriction ensures a fair comparison across all spectral regions, as using more PCs could potentially improve the performance and bias the evaluation in favor of spectral region B. However, in each case, the number of significant PCs allows us to explain approximately 80% of the total variance. RMSE values were calculated by using the mentioned number of PCs. For all spectral regions, the RMSE values ranged from 0.0029 to 0.0031, indicating a comparable performance across all regions to capture the inherent variance within the data. Linear and nonlinear SVM models were trained using the same number of PCs. Across all models, sensitivity values were generally 5–9% lower than the respective specificity values, which is expected in multiclass classification tasks with an unbalanced data distribution [54]. To mitigate data imbalance, we applied random undersampling of majority classes to ensure a balanced dataset. Synthetic oversampling strategies, such as the synthetic minority oversampling technique applied to Raman spectra to increase the data size in minority classes, may further improve performance in future studies [55]. We used a one-against-one strategy for multiclass SVM, which is more effective for smaller datasets and lower class numbers, and promises higher accuracy for nonlinear SVM [56]. Sensitivity and specificity are strongly correlated by the choice of one-against-one or one-against-all classification in case of multiclass SVM. Interestingly, quadratic SVM did not outperform linear SVM, aligning with findings by Han et al. [57], who reported increased overfitting risks with nonlinear kernels, especially when the number of features (*m*) approaches or exceeds the number of samples (*n*) [58]. Our feature space was reduced via PCA, resulting in *m*≪*n* for all spectral selections, thereby minimizing the overfitting risk and aligning with established guidelines on feature-to-sample ratios for robust classifier training. Permutation analysis in combination with PCA-based SVM allows for a reasonable feature selection and minimizes the risk of overfitting. Still, performance differences may also be attributed to class overlap, which slows and weakens SVM learning [42]. Simple linear kernels demonstrated similar performance in case of class overlap. For example, PCA score plots (Figure 4) showed a considerable overlap in spectral region B, which correlated with the lowest classification performance among all regions. Although spectral region C showed the best PCA-based variance explanations, the best-performing linear SVM model was trained on spectral region A. This could be due to the reduced overlap across the first 15PCs, which were used in training. In contrast, quadratic SVM models exhibited complex behavior due to the trade-off between better boundary decision making and increased sensitivity to overlapping and overfitting.

Despite its diagnostic potential, RS inherently requires long acquisition times due to its weak signal cross sections. Our LSRM approach reduces acquisition time, but speed still remains a limitation for real-time clinical use. The small FOV, high sensitivity to motion artifacts, and ambient light present challenges, especially in surgical environments. An endoscopic implementation of RS for fibrotic tissue detection could therefore benefit from the proposed Raman band selection. Narrowing the spectral acquisition window to region C (1400–1750 cm^−1^) allows us to reduce the systems’ complexity while maintaining key biochemical information about CH_2_ and CH_3_ deformation as well as amide I, all of which are related to collagen content. A limited spectral range reduces the requirements for optical filtering and allows for smaller detector arrays with faster acquisition rates. In addition to the compact hardware, computational efforts are also reduced that way. The constant motion of the heart still challenges a motion-free tissue contact required for sufficient Raman signal collection. In recent years, pharmacological treatments with adenosine have been established as a straightforward technique to induce transient standstill with asystole durations up to 44 s [59,60]. With this treatment, the Raman signal collection can be prolonged to obtain sufficient molecular information within the reduced spectral range. These combined advances might pave the way for Raman endoscopes that offer molecular-specific guidance during surgical or electrophysiological procedures. As an alternative, faster Raman modalities such as stimulated Raman scattering (SRS) may be used. SRS enables rapid acquisition with a limited spectral range, which might be suitable for fast pathological cardiac tissue screening in combination with dimensionality reduction, regression analysis, and ML [61]. Recent developments in endoscopic SRS and multimodal optical imaging probes offer viable pathways for integration into clinical workflows [62]. Optical coherence tomography (OCT) is another complementary non-invasive technique that provides real-time imaging of cardiac microstructures with ultrahigh resolution based on changes in the refractive index [63]. A combined OCT-SRS platform targeting collagen-specific Raman bands at $1448\;{\mathrm{cm}}^{-1}$ and $1652\;{\mathrm{cm}}^{-1}$ could offer complimentary information about morphological and molecular changes in a non-invasive and label-free manner. Such multimodal probes combining OCT, SRS, and electrophysiological techniques have the potential to significantly improve the sensitivity to arrhythmogenic substrates following MI.

## 4. Materials and Methods

### 4.1. Sample Preparation and Masson’s Trichrome Staining

This experimental study included hearts that were obtained from an ovine chronic myocardial infarction model (N = 2 sheep, 1 year old at the time of surgery and 40–50 kg). Protocol was accepted by the Animal Research Ethics Committee (Comité d’Ethique en Expérimentation Animale de Bordeaux; CEEA50) at the University of Bordeaux, France, and approved by the Directorate-General for Research and Innovation, Unit for Animals Used for Scientific Purposes (Direction générale de la recherche et de l’innovation, Cellule Animaux utilisés à des Fins Scientifiques-AFiS), in accordance with the European rules for animal experimentation (European legislation 2010/63/UE; 2010). A sample size of two was chosen to adequately demonstrate sample variability with maximum reduction in animal usage. Animals were considered healthy at the time of enrollment and no specific exclusion criteria were applied. We did not control for confounding factors and all animals received the same experimental procedures. The ovine infarction was induced as described by Dib et al. [64]. Sheep were premedicated with ketamine (20 mg/kg, intramuscular) and acepromazine (0.02 mL/kg, intramuscular). Anesthesia was induced by propofol (2 mg/Kg) before intratracheal intubation and maintained under isoflurane, 2%, in air/O2 (50/50%) [65,66]. A continuous intravenous infusion of antiarrhythmics and -blockers (Amiodarone 1–2 mg/kg/hour, Lidocaine 7–10 mg/kg, Magnesium 20 mg/kg/hour, Esmolol 2–6 mg/kg/hour) was administered. Clot formation was avoided by administration of herparin 0.5 mg/kg. A catheter was inserted through the femoral artery to the left ventricle. By deploying embolization coils (Boston Scientific Corporation, Marlborough, MA, USA) into the left anterior descending artery, the infarction was induced. After completion of the procedure, infusion of antiarrhythmic drugs, beta-blockers, and application of anesthetic molecules under oxygen was arrested and animals were extubated and transferred to a postoperative care facility, receiving antibiotics (amoxicillin 15 mg/kg IM) for 8 days and anti-inflammatories (Flunixin 2 mg/kg/day) for 4 days. The sheep were given a healing period of 6 weeks, and in this time frame, the scarring process occurred on the ischemic part of the left ventricle, with a scar formation around the coronary artery at the distal point of the embolization. After 6 weeks, the sheep were again premedicated and maintained as described above. Heparin (2 mg/kg) was injected intravenously to prevent blood coagulation before euthanasia by intravenous injection of pentobarbital (30 mL/50 kg), and the hearts were rapidly excised and flushed at 4 °C with a cardioplegic solution containing the following (mM): NaCl, 110; CaCl_2_, 1.2; KCl, 16; MgCL_2_, 16; NaHCO_3_, 10; and glucose, 9.01. Sheep hearts were perfused for 2 h with phosphate-buffered saline (PBS) containing EDTA to chelate calcium and inhibit contraction. Subsequently, the hearts were fixed by perfusion with 4% paraformaldehyde for 1 h, rinsed with PBS, and stored in PBS supplemented with Na-azide. Using a vibratome (7000 smz^−2^, Campden Instruments Ltd., Loughborough, Leicestershire, UK), the left ventricles were dissected from the harvested hearts and 400 μm thick slices covering an area of about 20mm×25mm from different locations from the left ventricles, especially around the infarction area, were used for all experiments. Slices were secured using two metal clips mounted on modeling clay to isolate the cardiac tissue from underlying surfaces. The clips were positioned near the edges of the slices to minimize the risk of tissue damage. The mounted tissue was put in a Petri dish and flooded with distilled water for immersion and to avoid dehydration of the biopsies. In total, three biopsies were obtained from the left ventricles of two sheep. Each biopsy was investigated at three distinct regions using our LSRM setup, resulting in a total of nine individual measurements. Each measurement was treated as a separate sample for subsequent analysis.

After imaging, the fixed tissue samples were embedded in paraffin and sectioned into 5 μm thin subslices parallel to the imaging planes, allowing us to link the histological information with the observed tissue composition, as shown in Figure 1. For histopathological classification, Masson’s TS was performed according to a standard protocol for identification of collagen-rich tissue (blue), muscle tissue (red), and nuclei (black). Slices were scanned using the Axio Scan Z.1 automated microscope slide scanner (Carl Zeiss AG, Oberkochen, Germany) and annotated by a trained pathologist into four different tissue types, namely muscle, necrotic, granulated, and fibrotic tissue.

### 4.2. Line Scan Microspectroscopy Setup

The LSRM system implemented in a custom-built upright laser scanning microscope (Eclipse E400, Nikon, Tokyo, Japan) is described elsewhere [67]. In brief, a customized continuous-wave Ti/Sapphire laser with a wavelength of 785nm was used for excitation. A combination of a Powell lens and a cylindrical lens (both Thorlabs, Newton, NJ, USA) was implemented to provide uniform line illumination. Galvanometric mirrors (6230H, Cambridge Technology, Bedford, MA, USA) were used to scan the focal plane. A three-axis motorized translation stage (X-LSM050A-E, Zaber, Vancouver, BC, Canada) was used to navigate through the specimen. LSRM was operated with a high-resolution objective (16x, CFI LWD Plan Fluorite Objective, 0.8 NA, Nikon, Japan) to achieve a lateral resolution of 10 μm and an efficient collection of Raman signals. The spectrometer’s spectral resolution was 0.5nm. Over the full line of illumination, the laser power on the sample plane was 80mW. A spectrometer (Shamrock SR 303i, Andor Technology, Belfast, Northern Ireland) with a 300L/mm grating blazed at 500nm was used for signal collection. The parameters of the spectrometer have been described elsewhere [68]. In short, the spectrometer had a spectral resolution of 0.5nm ($5.5\;{\mathrm{cm}}^{-1}$) and a slit size of 100 μm. The 255×1024pixel CCD camera (Newton 920i, Andor Technology, Belfast, Northern Ireland) was used to acquire LSRM spectra in binning mode, resulting in an acquisition area of 128×512pixels. A laser clean-up filter (785nm MaxLine^®^, LL01-785-12.5, Semrock, Rochester, NY, USA) and an ultra-steep long-pass edge filter (785nm RazorEdge^®^, LP02-785RE-25, Semrock, Rochester, NY, USA) were used to filter the excitation light. A long-pass dichroic mirror with a cut-on wavelength of 805nm (DMLP805, Thorlabs, Newton, NJ, USA) was used to separate the Raman signal from the excitation laser. All cardiac samples were scanned with a step size of 5 μm with the translational stage. For a wavenumber range between $550\;{\mathrm{cm}}^{-1}$ and $3626\;{\mathrm{cm}}^{-1}$, Raman spectra were collected simultaneously along the laser line, covering an area of 80 μm × 250 μm in 10min with a total amount of 51 stops. Each camera image was accumulated once with an acquisition time of 10s.

A combination of customized MATLAB 2024b and LabView 2023 Q1 software was developed to control the galvanometric mirrors and the motorized stage as well as to start excitation and acquisition.

### 4.3. Spectral Processing and Classification

In total, 14,229 spectra were collected, corresponding to 459 CCD camera acquisition files. All spectra were calibrated using a polystyrene reference standard [69]. The processing and analysis of the Raman data were performed using a custom MATLAB script following the protocol proposed by Bocklitz et al. [70]. The raw spectra covering the 600–2960 cm^−1^ wavenumber range were denoised using a smoothing spline fit [71]. Instrument-specific spectral responses, including lens, grating transmission, and CCD camera sensitivity, were corrected accordingly. To harmonize baseline variations, extended multiplicative signal correction was applied using an open-source toolbox [72]. Fluorescent background removal was performed using an iterative 9th polynomial fitting algorithm with a threshold of 0.1 [73]. The resulting baseline-corrected spectra were further smoothed with a Savitzky–Golay filter with a polynomial order of 5 and framelength of 51 [74]. The silent region between $1800\;{\mathrm{cm}}^{-1}$ and $2800\;{\mathrm{cm}}^{-1}$, which contains non-relevant molecular information, was excluded in the dataset. For each CCD acquisition, the spectra were averaged. Spectra were then grouped according to their respective histopathological classification and filtering was applied for each class individually. A spectrum was removed from the dataset of its class if more than 5% of its value deviated beyond a ±2 standard deviation from the class mean. After filtering, spectra from all classes were combined into a single dataset, resulting in 374 spectra. Each spectrum was L2-vector-normalized prior to further analysis [75]. The full processing workflow is provided in the Supplemental Materials in Figure S1.

For ratio-based analysis, peak intensities at $1448\;{\mathrm{cm}}^{-1}$ and $1652\;{\mathrm{cm}}^{-1}$ were extracted. The resulting intensity ratios were confirmed to follow a normal distribution for each class. A one-way ANOVA was performed to determine statistically significant differences among the histopathological classes. All processing and analysis steps, as well as dimensionality reduction—including linear and nonlinear dimension reduction techniques for discrimination, including PCA, nonlinear PCA, and SVM—were performed in MATLAB. For nonlinear PCA, an open-source autoencoder-based algorithm was used [29]. Prior to SVM training, data-balancing was applied to the filtered spectra to avoid class imbalance. The smallest class size was used as a reference, and an equal number of samples were randomly sampled from the larger classes, resulting in a final balanced dataset of 320 spectra. Study objectives were to validate technological developments requiring only example tissue samples from cardiac disease. Therefore, all study comparisons were between technological approaches, rather than experimental groups. Each sample was labeled according to one of the four predefined histopathological categories: muscle, necrotic, granulated, or fibrotic tissue. Linear PCA was performed on three different spectral regions (A, B, C) and the amount of PCs used for subsequent SVM training was determined by two criteria: firstly, the selected PCs explain approximately 80% of the total variance, and secondly, the cumulative variance across all spectral regions are as comparable as possible to ensure fair validation. Linear and quadratic SVM classifiers were trained using the selected PCs for each spectral band selection. A five-fold cross-validation scheme was applied, and a one-versus-one classification strategy was used to accommodate the multiclass setting. Classification performance was evaluated using total sensitivity and specificity as the mean values across all four classes.

To assess the statistical reliability and independence of the variables, a permutation analysis was conducted on the multiclass linear PCA results [76]. Following the null hypothesis of no significant association existing between features and class labels, all variables were randomly sampled 1000 times. For each permutation, a linear PCA model was computed and the explained variances of all components were compared to those of the original model. P-values were calculated by counting the number of permutations where the variance explained by a given PC in the permuted model exceeded the variance of the original PC. This count was divided by the total number of permutations to assess the statistical reliability of each PC.

## 5. Conclusions

Our study demonstrates the potential of LSRM as a label-free imaging modality with high spatial resolution for the classification of myocardial tissue across various stages of fibrotic remodeling. By focusing on two prominent collagen-associated Raman peaks at $1448\;{\mathrm{cm}}^{-1}$ and $1652\;{\mathrm{cm}}^{-1}$, we were able to distinguish between muscle, necrotic, granulated, and fibrotic tissue, validated through Masson’s TS. These findings underscore the molecular sensitivity of RS in detecting collagen-related changes that accompany MI and scar formation. Our comparative evaluation of different spectral bands revealed that the spectral region C, with wavenumbers between $1400\;{\mathrm{cm}}^{-1}$ and $1750\;{\mathrm{cm}}^{-1}$, dominated by strong collagen signatures provided the highest information density, explained variance, and clustering performance in both binary and multiclass classification. Despite the nonlinear nature of fibrotic scarring, nonlinear PCA offered only marginal improvements over standard linear PCA. Similarly, quadratic SVM models did not consistently outperform their linear counterparts, likely due to increased sensitivity to class overlap and overfitting in high-dimensional data. Our approach illustrates that a targeted spectral analysis focusing on biologically relevant molecular signatures can enhance tissue discrimination for potential intraoperative applications. Furthermore, we show that a balanced integration of dimensionality reduction and ML algorithms, supported by permutation-based feature selection, allows for robust classification performance while minimizing overfitting risk even with limited spectral data and sample size. In summary, LSRM combined with advanced data analysis offers a promising route for high-resolution fibrosis detection and extension of conventional electrophysiological diagnostics, with the potential to improve treatment outcomes in MI and beyond.

## Figures and Tables

**Table 1:** Explained variance for the first three PCs for different spectral regions.

Principal Component	Spectral Region A	Spectral Region B	Spectral Region C
First	32.2%	26.8%	57.8%
Second	6.3%	7.4%	7.4%
Third	5.4%	6.5%	3.3%
Sum	43.9%	40.7%	68.5%

**Table 2:** Percentage of total variance explained by the first two PCs for each Raman spectral region, comparing linear and nonlinear PCA models.

Principal	Spectral Region A		Spectral Region B		Spectral Region C	
Component	Linear PCA	Nonlinear PCA	Linear PCA	Nonlinear PCA	Linear PCA	Nonlinear PCA
First	45.7%	48.4%	37.4%	40.3%	72.6%	74.6%
Second	4.6%	3.7%	5.8%	5.7%	4.7%	4.1%
Sum	50.3%	52.1%	43.2%	46.0%	77.3%	78.7%

**Table 3:** Number of PCs used for SVM training, along with the corresponding explained variance and RMSE values from PCA reconstruction. Performance metrics of trained linear and quadratic SVM models for tissue classification are shown for different Raman spectral regions.

	Spectral Region A		Spectral Region B		Spectral Region C	
	Linear SVM	Quadratic SVM	Linear SVM	Quadratic SVM	Linear SVM	Quadratic SVM
Number of PCs	15		15		3	
Explained Variance	79.2%		79.9%		79.7%	
RMSE	0.0030		0.0029		0.0031	
Total Sensitivity	90.9%	87.2%	88.8%	86.9%	89.4%	89.1%
Total Specificity	97.0%	95.7%	96.3%	95.6%	96.5%	96.4%

## Data Availability

Data available upon reasonable request.

## Supplementary Materials

The following supporting information can be downloaded at https://www.mdpi.com/article/10.3390/ijms26157240/s1.

## Abbreviations

The following abbreviations are used in this manuscript:

ANOVA	Analysis of Variance
AUC	Area under the ROC Curve
ECM	Extracellular Matrix
FOV	Field of View
LSRM	Line Scan Raman Microspectroscopy
MI	Myocardial Infarction
ML	Machine Learning
OCT	Optical Coherence Tomography
PBS	Phosphate-buffered Saline
PC	Principal Component
PCA	Principal Component Analysis
ROC	Receiver Operating Characteristic
ROI	Region of Interest
RS	Raman Spectroscopy
SCD	Sudden Cardiac Death
SRS	Stimulated Raman Scattering
SVM	Support Vector Machine
TS	Trichrome Staining
VF	Ventricular Fibrillation
VT	Ventricular Tachycardia
