# Supplementary material for: Spectral Region Optimization and Machine Learning-Based Nonlinear Spectral Analysis for Raman Detection of Cardiac Fibrosis Following Myocardial Infarction
Source: Int J Mol Sci. 2025 Jul 26;26(15):7240. doi: 10.3390/ijms26157240 (PMC12346871; doi:10.3390/ijms26157240)
Supplement: Supplementary file 1 [file ijms-26-07240-s001.zip › ijms-3698328-supplementary.pdf]

Supplemental Materials

Spectral Region Optimization and Machine Learning-Based Nonlinear Spectral Analysis for Raman Detection of Cardiac Fibrosis Following Myocardial Infarction

Arno Krause[1‡], Marco Andreana[1‡], Richard D. Walton[2], James Marchant[2], Nestor Pallares-Lupon[2], Kanchan Kulkarni[2], Wolfgang Drexler[1], Angelika Unterhuber[1*]

[1] Center for Medical Physics and Biomedical Engineering, Medical University of Vienna, Waehringer Guertel 18-20, 1090 Vienna, Austria

[2] IHU Liryc, Univ. Bordeaux, INSERM U 1045, CRCTB, F-33000 Bordeaux, France

[‡] These authors contributed equally to this work.

* Correspondence: angelika.unterhuber@meduniwien.ac.at

Contents

1 Raman Spectrum Processing **S2**

2 Alternative Raman band selection **S4**

2.1 Binary PCA S4

2.2 Quadruple linear and nonlinear PCA S6

2.3 Variance Explanations and PCA Model Performance S7

1 Raman Spectrum Processing

The processing of the Raman data was implemented in a custom MATLAB script following the protocol proposed by *Bocklitz et al.*[1]. The relevant steps are illustrated in Figure S1 for representative samples of each tissue group.

1. Raw spectra were cropped to the $600\,\mathrm{cm}^{-1}$ – $2960\,\mathrm{cm}^{-1}$ wavenumber range and denoised using a smoothing spline fit without washing out low-contrast peaks [2].

2. Instrument-specific spectral responses, such as optics and grating transmission, and CCD camera sensitivity, were corrected for each spectrum.

3. Extended Multiplicative Signal Correction (EMSC) was applied using an open-source toolbox [3].

4. For baseline correction and removal of the fluorescent background, an iterative 9th polynomial fitting algorithm with a threshold of 0.1 was used as proposed by *Lieber et al.*[4]. Afterwards, the silent region between $1800\,\mathrm{cm}^{-1}$ and $2800\,\mathrm{cm}^{-1}$ was excluded from each spectrum.

5. Further smoothing was performed with a Savitzky-Golay filter with a polynomial order of 5 and framelength of 51 [5].

6. Finally, each spectrum was normalized using L2 vector prior to further analysis [6].

In our study, we implemented this data processing pipeline with a two stage normalization for raw and fitted spectra to minimize influences of post-processing algorithms applied to Raman peak intensities and to improve the robustness for further analysis of intensity-based comparisons.
For the RMSE calculations, the spectral data was reconstructed using the score and loading (coefficient) matrices of a PCA model. The reconstructed spectral data R is given by

$$R = S \cdot C^{\top} + \mu, \tag{1}$$

where S is a $n \times k$ matrix, C is a $m \times k$ matrix, and μ is a vector with size $1 \times m$ containing the mean of all intensity values for a wavenumber. Since the input data during PCA is typically mean-centered, the mean vector μ is added after reconstruction. Further, n is the number of spectra, m is the number of wavenumbers, and k is the number of used PCs. All PCs required to explain approximately 80 % of the total variance were included. The RMSE value is then obtained by calculating the deviations between original intensity values I_i and reconstructed intensity values R_i for all N Raman intensity values:

$$\mathrm{RMSE} = \sqrt{\frac{1}{N}\sum_{i=1}^{N}(I_i - R_i)^2}. \tag{2}$$

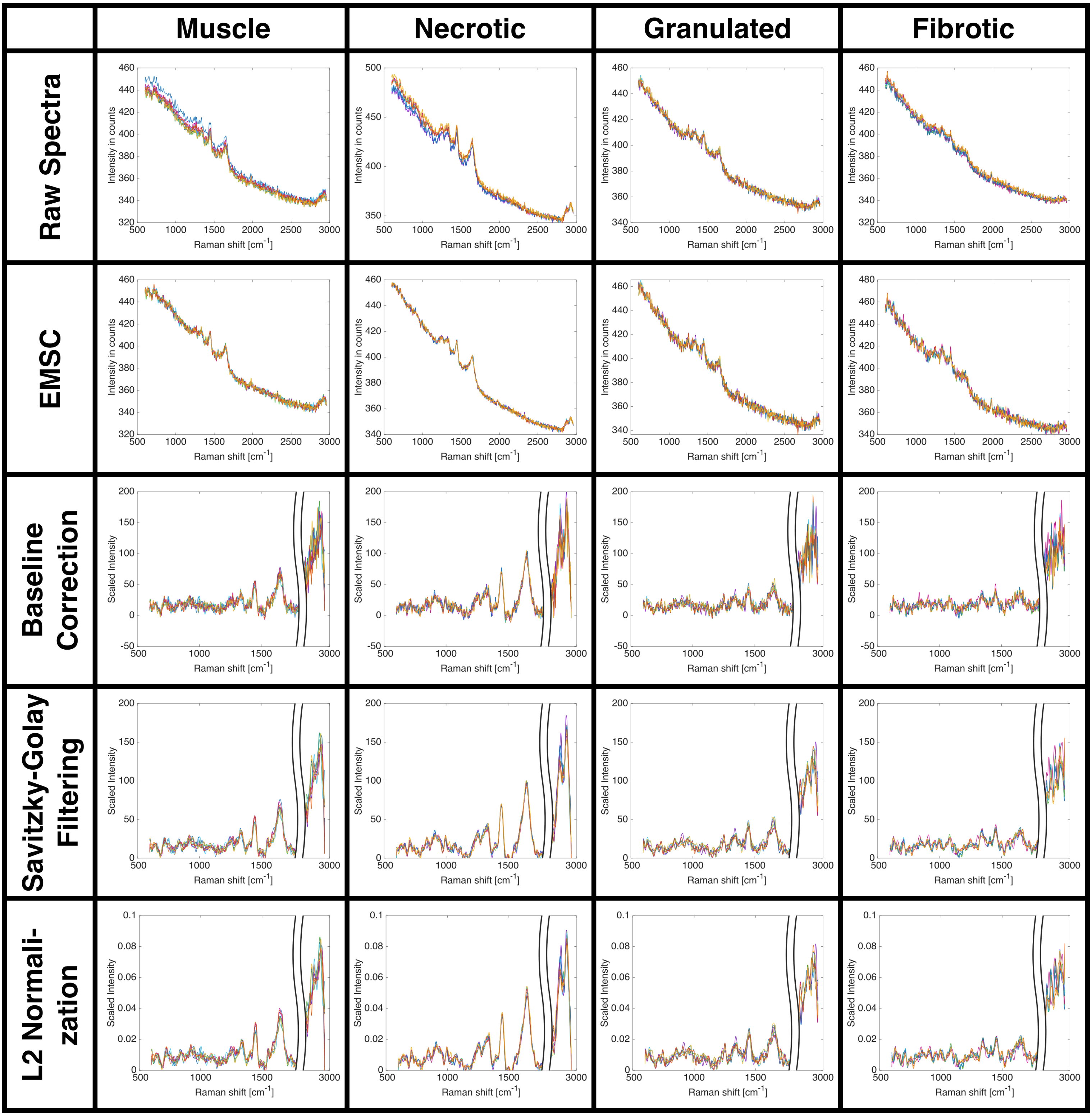

Figure S1: Post-processing steps for representative spectral data of each tissue group. All steps are implemented in a custom MATLAB script

2 Alternative Raman band selection

Studies on spectral range optimization highly depend on the choice of the spectral segments used for analysis. In our study, we focused on the following spectral regions: region A ($600\,\mathrm{cm}^{-1} - 2960\,\mathrm{cm}^{-1}$), region B ($600\,\mathrm{cm}^{-1} - 1399\,\mathrm{cm}^{-1}$ and $1751\,\mathrm{cm}^{-1} - 2960\,\mathrm{cm}^{-1}$), and region C ($1400\,\mathrm{cm}^{-1} - 1750\,\mathrm{cm}^{-1}$). Region A covers the fingerprint and C-H stretching region to obtain all relevant molecular information. Region B provides information about the full molecular fingerprint region excluding region C. Region C offers information about a specific Raman band including collagen-associated peaks ($1448\,\mathrm{cm}^{-1}$ and $1652\,\mathrm{cm}^{-1}$), which leveraged to powerful biomarkers for the classification and staging of myocardial fibrosis. For completeness basic analysis was also performed on region D ($600\,\mathrm{cm}^{-1} - 1750\,\mathrm{cm}^{-1}$) and E ($1750\,\mathrm{cm}^{-1} - 2960\,\mathrm{cm}^{-1}$).
Similar to region C, region D contains the collagen-associated peaks at $1448\,\mathrm{cm}^{-1}$ and $1652\,\mathrm{cm}^{-1}$, but includes further collagen-associated peaks, such as the vibrational band at $1252\,\mathrm{cm}^{-1}$ [7]. Region E covers the C-H stretching region. In the following, the binary PCA using muscle and fibrotic tissue, and the linear and nonlinear PCA using all four tissue classes are presented for regions D and E.

2.1 Binary PCA

The mean Raman spectrum in Figure S2 c visualizes the Raman bands for spectral regions D and E. The score plots of the first two PCs of spectral regions D and E are shown in Figure S2 a and b, respectively. Class separation is observed along the first PC. Spectral region D in Figure S2 a shows comparable performance compared to region C in terms of class separation between fibrotic and muscle tissue. However, the additional wavenumber range taken from the fingerprint region causes a subclustering of single samples in both, muscle and fibrotic tissue. Such behavior is not desired in the context of binary classification, where clear and simple separation between the two classes is preferred. For region E shown in Figure S2 b, no distinct clustering between muscle and fibrotic tissue can be observed and the two clouds show a large overlap. Moreover, the score plot of region E demonstrates the complex relation between cluster performance and variance explanations. Although spectral region E has a relatively high explained variance (s. Table S1) and shows the steepest increase of the accumulated variance (s. Figure S2 d), the clustering performance is poor. A higher explained variance does not necessarily result in a better model [8]. A more detailed discussion is provided in Section 2.3. In our study, the explained variance for the first three PCs are highest for spectral region C (68.5 %), followed by region E (53.0 %) and region D (49.8 %) shown in Table S1.

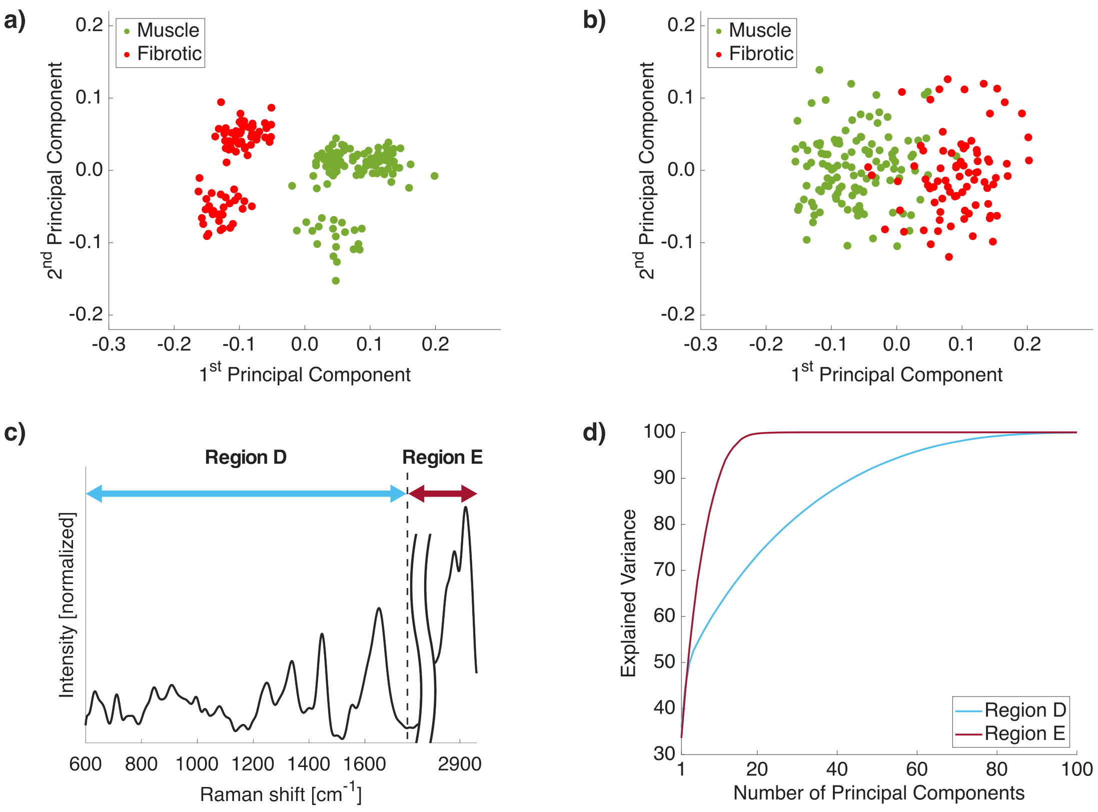

Figure S2: Score plots from linear PCA applied to (a) spectral region D ($600\,\mathrm{cm}^{-1}$ – $1750\,\mathrm{cm}^{-1}$), and (b) spectral region E ($1750\,\mathrm{cm}^{-1}$ – $2960\,\mathrm{cm}^{-1}$, excluding the silent region). (c) Mean Raman spectrum of all Raman spectra indicating the spectral coverage of regions D and E. (d) Cumulative explained variances.

Table S1: Explained variance for the first three PCs for spectral region D and E.

Principal Component	Spectral Region	
	D	E
First	36.0 %	33.6 %
Second	8.8 %	10.4 %
Third	5.0 %	9.0 %
Sum	49.8 %	53.0 %

2.2 Quadruple linear and nonlinear PCA

The multiclass linear and nonlinear PCA of regions D and E presented in Figure S3 show the same trend as the binary PCA. The clustering in region D is comparable to the clustering in region C and the four tissue types are clearly distinguishable. However, also a subclustering into single samples could be observed in this case. Region D has a cumulative variance for the first two PCs of 56.9 % in case of linear PCA and 58.2 % in case of nonlinear PCA, which are both below the variance explanations of region C, as shown in Table S2. Region E shows no clear class separation and has the worst clustering performance in the score plots. However, region E accounts for 54.5 % of the cumulative variance explanations using the first two PCs compared to region D with 52.2 %.

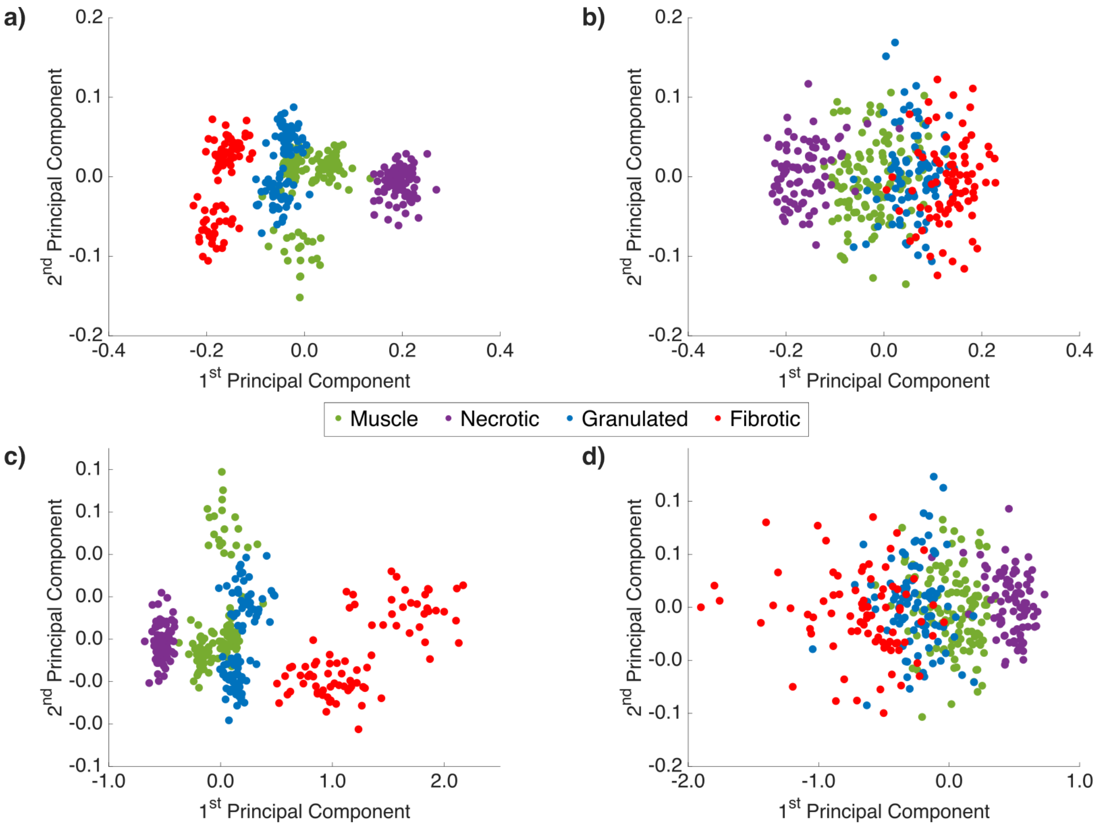

Figure S3: PC score plots derived from linear PCA applied to (a) spectral region D ($600\,cm^{-1} - 1750\,cm^{-1}$), and (b) spectral region E ($1750\,cm^{-1} - 2960\,cm^{-1}$, excluding the silent region). PC score plots derived from nonlinear PCA applied to (c) spectral region D, and (e) spectral region E.

Table S2: Percentage of total variance explained by the first two PCs for the Raman spectral regions D and E, comparing linear and nonlinear PCA models.

PC	Spectral region D		Spectral region E	
	linear PCA	nonlinear PCA	linear PCA	nonlinear PCA
First	51.2 %	53.0 %	44.1 %	16.1 %
Second	5.7 %	5.2 %	8.1 %	8.4 %
Sum	56.9 %	58.2 %	52.2 %	54.5 %

2.3 Variance Explanations and PCA Model Performance

PCA essentially identifies the directions of maximum variance in the data and aligns the PCs accordingly [9]. These new variables aim to preserve as much variability as possible. Consequently, when the dataset contains several variables (in our case, Raman wavenumbers) with high standard deviations across observations (in our case, Raman spectra), multiple PCs capture large amounts of the total variance. As a result, only a few PCs may be needed to explain, for example, 80 % of the cumulative variance. Therefore, the commonly held assumption that PCs with larger associated variances necessarily reflect meaningful or relevant dynamics within the data does not hold universally across all applications [10].

In our study, we observed this behavior as region E with high variances broadly distributed across many variables did not lead to meaningful and well-separated clusters in the resulting PCA score plots and did not allow for clear tissue classification. In contrast, region C with high standard deviation concentrated in a small number of variables provided superior cluster performance as shown in Figure S4. The shadowed areas showing the variations indicate that high variations are observed at peak maxima and minima rather than at side lobes. In addition, these peaks are associated to collagen, which is a key biomarker for cardiac fibrosis. As consequence, class separation in the PCA score plots follows the progress in fibrosis rather then general spectral variations. Since collagen-associated peaks at 1448 cm^{-1} and 1652 cm^{-1} are also included in region D, the classification outperforms region E (s. Figure S3), even though the cumulative explained variances are comparable (s. Table S2).

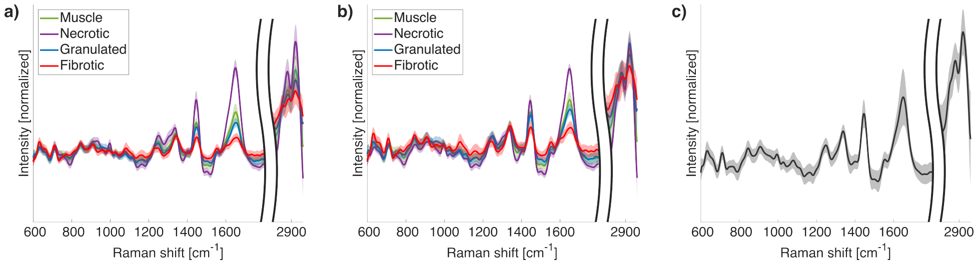

Figure S4: (a) Mean spectra of the four tissue classes: muscle, necrotic, granulated, and fibrotic tissue classes after filtering of outliers. (b) Mean spectra of the four tissue classes after filtering and L2 normalization. (c) Mean spectra of all four tissue classes after filtering and L2 normalization used for PCA. All shaded areas indicate the standard deviation across all spectra within each class.

Table S3: Root Mean Squared Error (RMSE) of the linear PCA models from the spectral regions D and E with an explained variance of about 80 %. For each spectral region, the number of PCs was calculated individually.

	Spectral Region	
	D	E
Number of PCs	23	7
Explained Variance	79.7 %	80.9 %
Root Mean Squared Error	0.0023	0.0052

A more reliable assessment of PCA model performance may be achieved by including absolutes measures, such as root mean square error (RMSE). In RMSE calculations, the original spectral data is reconstructed based on the scores and loadings of the PCA model for a given number of PCs. Comparing deviations between original and reconstructed Raman intensities for regions D and E lead to the RMSE values. Table S3 shows these error values for regions D and E of the corresponding linear PCA models. The number of PCs was set to explain 80 % of the variance to provide comparable conditions. Notably, the RMSE is not normalized, since the spectral regions were taken from same original data set. In fact, normalizing would possibly introduce unwanted bias, especially when peaks of interest differ in intensity or contrast across regions. Region E shows the highest RMSE, which aligns with the poor cluster performances, but high variance explanations. Region D shows a lower RMSE compared to regions A and C. PCA as unsupervised method identifies directions of maximum variance in the data rather than directions of class separation or biological relevance.
This investigations illustrate that relying solely on cumulative variance (e.g., selecting the minimum number of PCs that explain 80 % of the variance), or computational errors is not always a reliable strategy. Further performance metrics, such as distribution and relevance of variance across variables, cluster performance in the score plots, and RMSE benchmarking should be considered.

References

(1) Bocklitz, T.; Walter, A.; Hartmann, K.; Rösch, P.; Popp, J. *Analytica Chimica Acta* **2011**, *704*, 47–56.

(2) Green, P.; Silverman, B., *Nonparametric Regression and Generalized Linear Models: A roughness penalty approach*; Chapman & Hall/CRC Monographs on Statistics & Applied Probability; Taylor & Francis: 1993.

(3) Afseth, N. K.; Kohler, A. *Chemometrics and Intelligent Laboratory Systems* **2012**, *117*, Special Issue Section: Selected Papers from the 1st African-European Conference on Chemometrics, Rabat, Morocco, September 2010 Special Issue Section: Preprocessing methods Special Issue Section: Spectroscopic imaging, 92–99.

(4) Lieber, C. A.; Mahadevan-Jansen, A. *Applied Spectroscopy* **2003**, *57*, PMID: 14658149, 1363–1367.

(5) Savitzky, A.; Golay, M. J. E. *Analytical Chemistry* **1964**, *36*, 1627–1639.

(6) Guo, S.; Popp, J.; Bocklitz, T. *Nature Protocols* **2021**, *16*, 1–37.

(7) Cárcamo-Vega, J.; Aliaga, A.; Clavijo, E.; Manuel, B.; Vallette, M. *Journal of Raman Spectroscopy* **2012**, *43*, 248–254.

(8) Kjeldahl, K.; Bro, R. *Journal of Chemometrics* **2010**, *24*, 558–564.

(9) Jolliffe, I. T.; Cadima, J. *Philosophical transactions of the royal society A: Mathematical, Physical and Engineering Sciences* **2016**, *374*, 20150202.

(10) Shlens, J. *arXiv preprint arXiv:1404.1100* **2014**.
